# Surveillance of the liver in type 2 diabetes: important but unfeasible?

**DOI:** 10.1007/s00125-024-06087-7

**Published:** 2024-02-09

**Authors:** Sami Qadri, Hannele Yki-Järvinen

**Affiliations:** 1 Department of Medicine, University of Helsinki and Helsinki University Hospital, Helsinki, Finland; 2 Minerva Foundation Institute for Medical Research, Helsinki, Finland

**Keywords:** Biomarkers, Cirrhosis, Fatty liver, Fibrosis, NAFLD, NASH, Prevalence, Prognosis, Review, Screening, The metabolic syndrome, Type 2 diabetes

## Abstract

Fatty liver plays a pivotal role in the pathogenesis of the metabolic syndrome and type 2 diabetes. According to an updated classification, any individual with liver steatosis and one or more features of the metabolic syndrome, without excess alcohol consumption or other known causes of steatosis, has metabolic dysfunction-associated steatotic liver disease (MASLD). Up to 60–70% of all individuals with type 2 diabetes have MASLD. However, the prevalence of advanced liver fibrosis in type 2 diabetes remains uncertain, with reported estimates of 10–20% relying on imaging tests and likely overestimating the true prevalence. All stages of MASLD impact prognosis but fibrosis is the best predictor of all-cause and liver-related mortality risk. People with type 2 diabetes face a two- to threefold increase in the risk of liver-related death and hepatocellular carcinoma, with 1.3% progressing to severe liver disease over 7.7 years. Because reliable methods for detecting steatosis are lacking, MASLD mostly remains an incidental finding on imaging. Regardless, several medical societies advocate for universal screening of individuals with type 2 diabetes for advanced fibrosis. Proposed screening pathways involve annual calculation of the Fibrosis-4 (FIB-4) index, followed by a secondary test such as transient elastography (TE) for intermediate-to-high-risk individuals. However, owing to unsatisfactory biomarker specificity, these pathways are expected to channel approximately 40% of all individuals with type 2 diabetes to TE and 20% to tertiary care, with a false discovery rate of up to 80%, raising concerns about feasibility. There is thus an urgent need to develop more effective strategies for surveying the liver in type 2 diabetes. Nonetheless, weight loss through lifestyle changes, pharmacotherapy or bariatric surgery remains the cornerstone of management, proving highly effective not only for metabolic comorbidities but also for MASLD. Emerging evidence suggests that fibrosis biomarkers may serve as tools for risk-based targeting of weight-loss interventions and potentially for monitoring response to therapy.

**Graphical Abstract:**

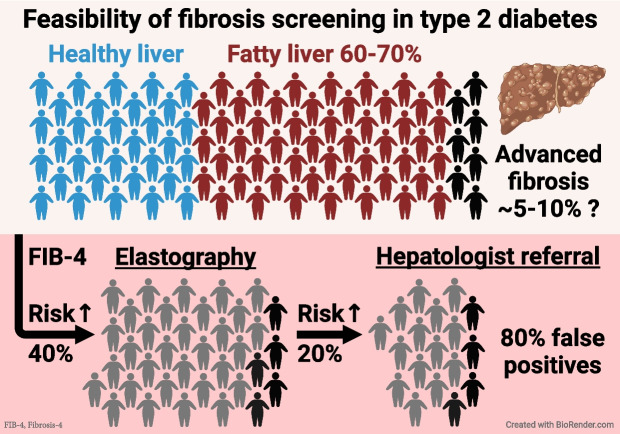

**Supplementary Information:**

The online version contains a slideset of the figures for download available at 10.1007/s00125-024-06087-7.


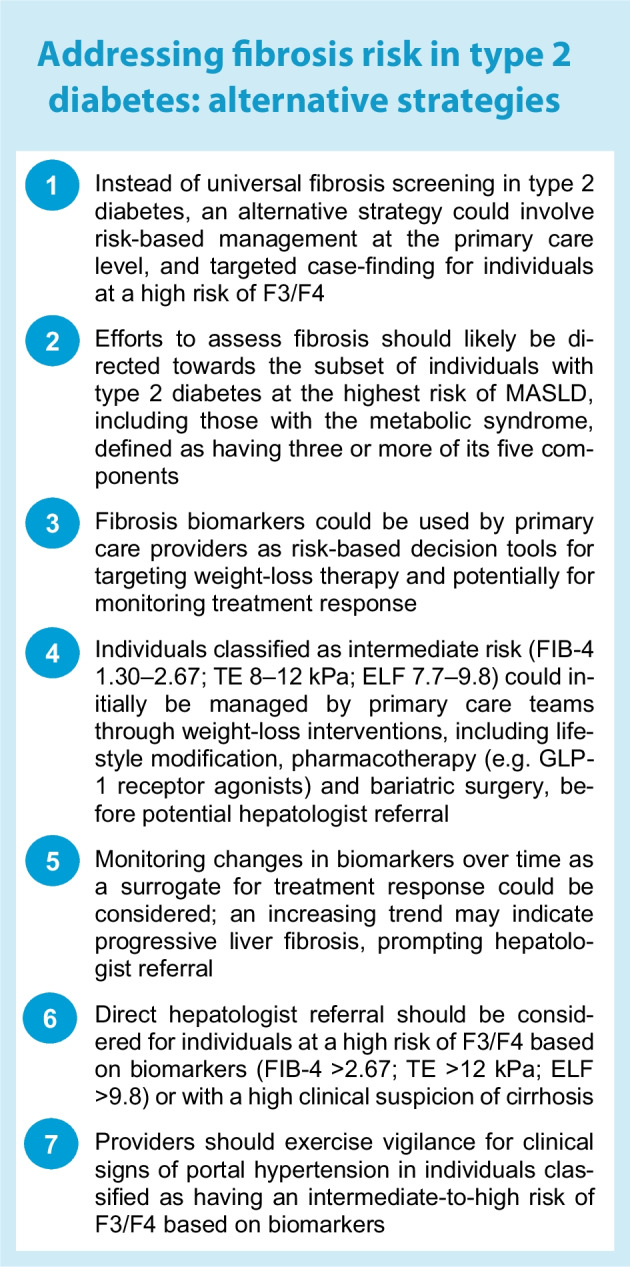



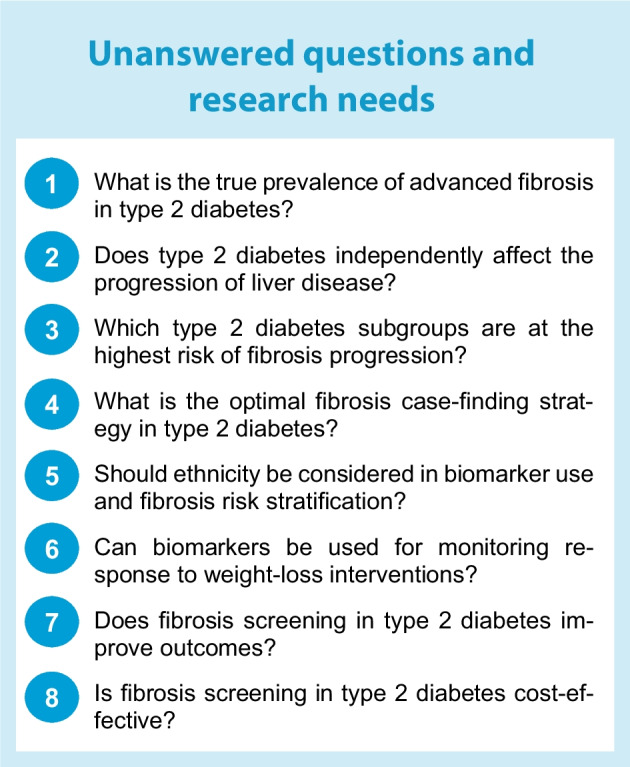


## Introduction

The liver is a central organ in the pathogenesis of type 2 diabetes. After an overnight fast, it produces all of the circulating glucose, as described by Claude Bernard already in the mid-1850s [1]. This process is tightly controlled by both insulin and glucagon. Postprandially, insulin suppresses hepatic glucose production, a function compromised in type 2 diabetes, which was first documented by Dame Sheila Sherlock and colleagues in 1951 using hepatic venous catheterisation [2]. Subsequent studies confirmed this as the key defect underlying postprandial hyperglycaemia in type 2 diabetes [3]. Indeed, glucose use in insulin-dependent tissues such as muscle remains normal, as hyperglycaemia compensates for any defect in peripheral insulin action [4]. In addition to glucose, the insulin-resistant liver overproduces VLDL, contributing to fasting hypertriglyceridaemia and ultimately to a decreased HDL-cholesterol concentration [5].

Non-invasive quantification of liver fat content by imaging, such as proton magnetic resonance spectroscopy (^1^H-MRS), and using this as a basis to rank individuals, has revealed that hepatic insulin resistance of both glucose [6] and VLDL production [7] are directly related to liver fat content. Thus, the pathogenesis of type 2 diabetes is intertwined with that of the steatotic liver. Accumulation of hepatic triglyceride occurs in most individuals due to excess energy intake, independent of alcohol consumption [8, 9]. This type of liver steatosis has therefore been called non-alcoholic fatty liver disease (NAFLD). Steatosis is not just a marker of metabolic abnormalities, however; it also precedes and predicts progressive liver disease [10]. During the past 30 years, NAFLD has emerged as the most common liver disorder worldwide and a significant cause of end-stage liver disease [11]. Recently, as discussed below, new nomenclature and diagnostic criteria were introduced to replace the NAFLD classification [12].

This discussion begins by briefly reviewing the pathogenesis of liver disease in the context of type 2 diabetes. Next, we highlight a newly published update to the nomenclature and classification of steatotic liver disease. This is followed by a critical review of data describing the prevalence of liver disease in type 2 diabetes and its impact on mortality risk. Lastly, we explore the implications of recent guidelines recommending universal screening for liver fibrosis in individuals with type 2 diabetes, evaluate the feasibility of these strategies and consider future directions.

## Why does the liver become damaged in type 2 diabetes?

**Fig. 1:**
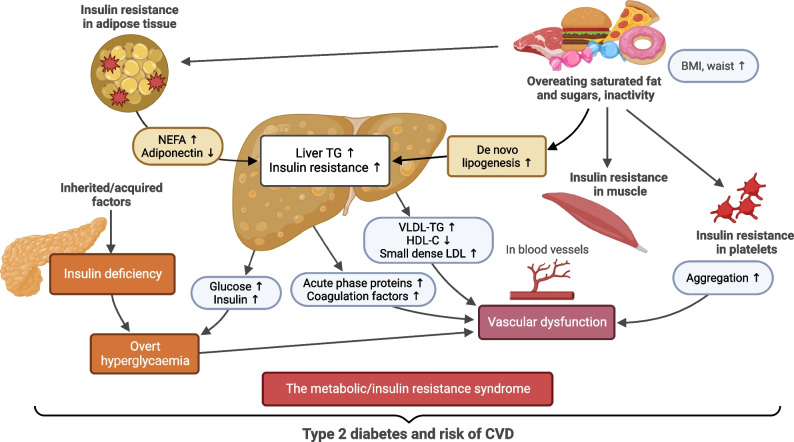
Pathogenesis of liver steatosis in type 2 diabetes. Overeating of especially saturated fat and simple sugars is the main reason for obesity and increased liver triglyceride synthesis. Fatty acids in hepatic triglycerides originate from the diet or adipose tissue lipolysis, or are synthesised in the liver de novo from precursors such as simple sugars and amino acids. Insulin resistance in adipose tissue is associated with decreased secretion of adiponectin and increased lipolysis, which drives steatosis by augmenting the flux of circulating NEFAs into the liver. Accumulation of liver triglycerides induces hepatic insulin resistance, leading to an overproduction of acute-phase proteins, coagulation factors, glucose and VLDL. The increased production of VLDL induces atherogenic dyslipidaemia, which is characterised by hypertriglyceridaemia and increased small dense LDL and decreased HDL-cholesterol concentrations. Insulin resistance in the liver, adipose tissue and muscle leads to hyperinsulinaemia, partly due to increased demand for pancreatic insulin secretion and partly due to impaired hepatic insulin clearance. These metabolic changes, together with abnormal platelet function, induce endothelial vascular dysfunction and CVD risk [13]. Failure of pancreatic beta cells to sustain chronic hyperinsulinaemia due to inherited and acquired factors leads to overt hyperglycaemia and type 2 diabetes [8]. HDL-C, HDL-cholesterol; TG, triglyceride. Created with BioRender.com. This figure is available as part of a downloadable slideset

**Fig. 2:**
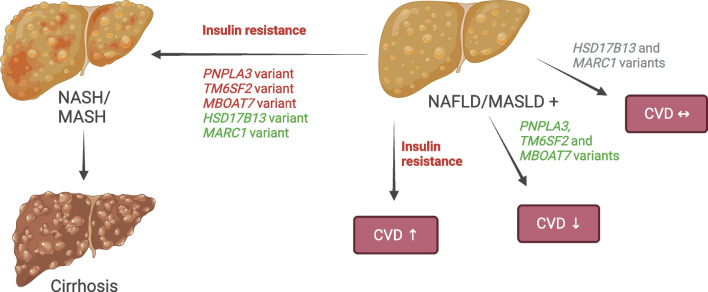
Consequences of steatosis. Insulin resistance predicts both severe liver disease and CVD [13]. In White populations, common genetic variants associated with NAFLD/MASLD predict >20% of the population-attributable fraction of chronic liver disease and >30% of the population-attributable fraction of cirrhosis [21]. Steatosis-predisposing variants in *PNPLA3*, *TM6SF2* and *MBOAT7* increase the risk of NASH/MASH and cirrhosis but paradoxically confer a protective effect against CVD [22, 23]. On the other hand, variants in *HSD17B13* and *MARC1* are protective against progressive liver disease but do not affect CVD risk. Red font denotes an adverse impact on outcomes; green font denotes a beneficial effect; grey font denotes a lack of impact on outcome. For discussion on the new definitions of MASLD and MASH, please refer to the section ‘Redefining steatotic liver disease: from NAFLD to MASLD’. Created with BioRender.com. This figure is available as part of a downloadable slideset

Numerous risk factors can aggravate liver steatosis in type 2 diabetes, encompassing (abdominal) obesity, excessive intake of saturated fat and simple sugars, and a sedentary lifestyle (Fig. 1) [8, 9]. Steatosis (i.e. abnormal triglyceride accumulation in hepatocytes) results from an influx of excess fatty acids into the liver from three main sources: (1) circulating fatty acids released through peripheral lipolysis; (2) de novo synthesis of fatty acids in the liver from lipogenic precursors; and (3) dietary intake of fatty acids [14–16]. Where liver triglyceride accumulation is highest, notably around the hypoxic terminal hepatic venules, hepatocytes may undergo ballooning necrosis [17]. This more active disease state of non-alcoholic steatohepatitis (NASH) may be accompanied by mild inflammation and varying degrees of fibrosis. The molecular mediators of hepatocellular damage are uncertain but could involve lipotoxic intermediates such as ceramides [18], which also characterise hepatic insulin resistance in humans [19]. The increased demand for insulin secretion explains why steatosis, independent of obesity, increases type 2 diabetes risk (Fig. 1). Insulin resistance-associated liver steatosis precedes and predicts NAFLD progression to NASH and advanced liver disease [13, 20] (Fig. 2). In addition, several common genetic polymorphisms explain, in European individuals, approximately 30% of the population-attributable risk of all stages of NAFLD, including cirrhosis [21] (Fig. 2). Inter-ethnic variations in NAFLD prevalence partly arise from differences in the population burden of common risk alleles [24]. While fibrosis in NAFLD tends to run in families [25], the extent to which this results from shared lifestyle vs genetic factors remains unclear.

## Redefining steatotic liver disease: from NAFLD to MASLD

The spectrum of NAFLD severity ranges from steatosis alone to NASH and cirrhosis [17]. NAFLD has been defined as steatosis unrelated to excess alcohol consumption (>20 g/day for women and >30 g/day for men) or other causes, as determined by careful family and medical history and potentially laboratory tests to exclude viral and autoimmune aetiologies and iron overload [26].

**Fig. 3:**
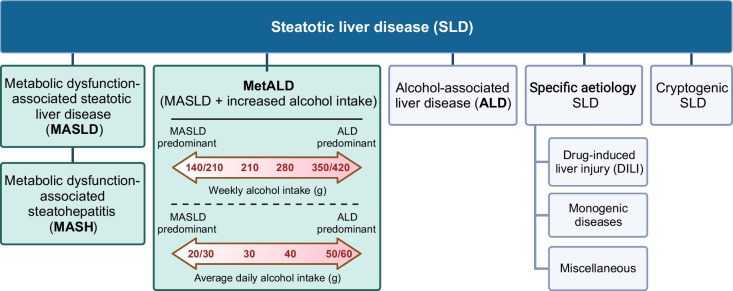
Updated classification of steatotic liver disease. In the diabetes clinic, steatotic liver disease is usually an incidental finding on abdominal imaging. MASLD is defined as hepatic steatosis in conjunction with one or more feature of the metabolic syndrome and no other discernible cause. If liver biopsy confirms histological inflammation and ballooning necrosis, the condition is termed MASH. The new classification considers that multiple aetiologies of steatosis can coexist, and that both metabolic dysfunction and significant alcohol use frequently contribute together to disease pathogenesis. Thus, metabolic dysfunction and alcohol-associated liver disease (MetALD) is defined as steatotic liver disease fulfilling the MASLD criteria in conjunction with an average alcohol intake of 20–50 g/day in women and 30–60 g/day in men (140–350 g/week in women and 210–420 g/week in men). Above these levels of alcohol consumption, alcohol-associated liver disease (ALD) could be considered to predominate. Other less common causes of steatotic liver disease (specific aetiology SLD and cryptogenic SLD) should be considered separately. Examples of monogenic diseases include lysosomal acid lipase deficiency, Wilson disease, hypoalphalipoproteinaemia and inborn errors of metabolism. Examples of miscellaneous disorders include hepatitis C virus, malnutrition and coeliac disease. Adapted from [12] with permission from Elsevier. Created with BioRender.com. This figure is available as part of a downloadable slideset

Given the frequent coexistence of NAFLD with the metabolic syndrome and type 2 diabetes, there has been a call to redefine the nomenclature and diagnostic criteria to better acknowledge this association. In 2020, a significant advance occurred when a group of experts initially proposed the definition of ‘metabolic dysfunction-associated fatty liver disease’ [27]. More recently, a large consensus group comprising content experts, practitioners and patient advocates published a similar albeit slightly modified classification update, endorsed by diverse liver organisations around the world [12]. According to this updated classification (Fig. 3), any individual with liver steatosis and even a single feature of the metabolic syndrome, without excess alcohol intake or other known causes of steatosis, has metabolic dysfunction-associated steatotic liver disease (MASLD). Accordingly, if liver biopsy confirms the presence of NASH, it is termed metabolic dysfunction-associated steatohepatitis (MASH), potentially accompanied by fibrosis ranging from stage F1 to F4 (F4 indicating cirrhosis).

While NAFLD and MASLD generally refer to the same condition [28], a key distinction is that a NAFLD diagnosis excludes other liver diseases, whereas MASLD is based on affirmative criteria. The new nomenclature also introduces a diagnostic category, metabolic dysfunction and alcohol-associated liver disease (MetALD), applying to individuals meeting MASLD criteria with concurrent excessive alcohol intake (as defined above) [12]. Moreover, this affirmative method of diagnosis now allows for the coexistence of other liver diseases with MASLD, such as autoimmune or viral hepatitis. Thus, in terms of disease classification, MASLD certainly seems like a step in the right direction. A potential concern with the new definition, however, is whether a single feature of the metabolic syndrome suffices to constitute ‘metabolic dysfunction’. For instance, elevated BP is common and has a strong age association, with a prevalence of ~80% among adults aged ≥65 years [29]. This contrasts with only a ~30% prevalence of hypertriglyceridaemia in those aged ≥60 years [30]. While MASLD is undoubtedly sensitive for metabolic dysfunction, whether the definition is specific enough to solely capture individuals with the intended aetiology, especially in the older population, warrants investigation.

Hereafter, this review will use MASLD in place of NAFLD, recognising that most referenced studies will have used the old definition.

## How common are features of MASLD in individuals with type 2 diabetes?

**Steatosis** Histologically, MASLD is characterised by at least 5% of hepatocytes containing predominantly macrovesicular (i.e. large) lipid droplets [17]. Steatosis can be quantified non-invasively via the proton density fat fraction (PDFF) by either ^1^H-MRS or MRI, as is commonly done in research settings but not in the clinical setting [31]. A prospective study in the USA used MRI–PDFF to measure liver fat in 501 adults with type 2 diabetes and found the prevalence of steatosis (PDFF >5% [31]) to be 65% [32]. Similarly, a meta-analysis estimated the prevalence of MASLD in type 2 diabetes to be 55% globally, rising to 68% in Europe [33]. These data suggest that most (60–70%) but not all individuals with type 2 diabetes have MASLD. Those without MASLD have been suggested to have diabetes subtypes that are not as closely linked to the metabolic syndrome but remain classified as type 2 diabetes [34].

### Steatohepatitis and fibrosis

The requirement of a liver biopsy to diagnose MASH renders estimation of its prevalence in type 2 diabetes difficult [33]. On the other hand, magnetic resonance elastography (MRE) and ultrasound-based transient elastography (TE) enable non-invasive assessment of liver fibrosis. MRE is still largely a research tool while TE is routinely used in hepatology clinics. Studies leveraging these methods to estimate the prevalence of advanced liver fibrosis (bridging fibrosis [stage F3] or cirrhosis [stage F4]) in type 2 diabetes often suggest it to be common: in excess of one in ten individuals (Table 1). The rate of fibrosis progression was recently examined in a paired-biopsy cohort of 208 individuals with MASLD and type 2 diabetes (79% with MASH, mean age 53 years, mean BMI 36 kg/m^2^) who had varying degrees of baseline fibrosis [49]. Over a median biopsy interval of 2.8 years, fibrosis progressed in 32%, remained unchanged in 44% and regressed in 24% of the study participants. The mean fibrosis progression rate was 0.23 stages per year in participants with a baseline fibrosis stage of F0 or F1 (i.e. approximately one stage per 4 years) and this was significantly higher than in participants without type 2 diabetes (0.16 stages per year) after adjustment for age, sex, race, ethnicity, BMI and baseline fibrosis stage [49]. A substantially lower progression rate (0.03 stages per year) was noted in placebo-treated participants (52.3% with type 2 diabetes) undergoing multiple per-protocol biopsies in MASH trials [50]. Because of inherent limitations related to histological fibrosis assessment it might, however, be more appropriate to evaluate disease progression via outcome-based hard endpoints such as mortality rate and incident severe liver disease [51]. To our knowledge, such data in type 2 diabetes populations are currently lacking.

**Table 1:** Summary of studies estimating the prevalence of advanced fibrosis in individuals with type 2 diabetes using non-invasive imaging tests

Study and location	Setting	*n*	F3/F4 definition	F3/F4 prevalence
Ajmera et al (2023), USA [32]	Family medicine and endocrinology clinics	493 (age ≥50 years)	MRE ≥3.63 kPa TE ≥8.8 kPa^a^	14.0%
Asero et al (2023), Italy [35]	Diabetes clinic	205	TE >10.1 kPa	15.6%
Chen et al (2020), Singapore [36]	Diabetes clinic	436	TE ≥9.6 kPa^b^	10.3%
Ciardullo et al (2021), USA [37]	Population-based study	825	TE ≥9.7 kPa	15.4%
Kang et al (2020), South Korea [38]	Health clinic	281	MRE ≥3.6 kPa	4.3%
Kwok et al (2016), Hong Kong [39]	Diabetes clinic	1884	TE ≥9.6 kPa^c^ TE ≥9.3 kPa^d^	17.7%
Lai et al (2019), Malaysia [40]	Diabetes clinic	557	TE ≥9.6 kPa^c^ TE ≥9.3 kPa^d^	21.0%
Lee et al (2023), Malaysia [41]	Diabetes clinic	258 (age ≥35 years and T2D duration ≥10 years)	TE ≥9.6 kPa^c^ TE ≥9.3 kPa^d^	22.1%
Lomonaco et al (2021), USA [42]	General internal medicine, endocrinology and family medicine clinics	561	TE ≥9.7 kPa	9%
Man et al (2023), China [43]	Nationwide study	411,409	TE ≥10.0 kPa	8.3%
Mantovani et al (2020), Italy [44]	Diabetes clinic	137	TE ≥8.7 kPa	10.2%
Mikolasevic et al (2020), Croatia [45]	Gastroenterology clinic	679	TE ≥9.6 kPa^c^ TE ≥9.3 kPa^d^	12.6%
Sporea et al (2020), Romania [46]	Diabetes clinic	534	TE ≥9.7 kPa	19.4%
Tuong et al (2020), Vietnam [47]	Health clinic	307	TE ≥8.7 kPa	5.9%
Wiafe et al (2023), Ghana [48]	Diabetes clinic	218	TE ≥8.9 kPa	5.6%

^a^TE was used if MRE was unavailable

^b^A controlled attenuation parameter ≥248 dB/m was used to define MASLD before TE assessment

^c^Cut-off used for TE M probe

^d^Cut-off used for TE XL probe

T2D, type 2 diabetes

## Validity of estimating fibrosis prevalence using imaging biomarkers

Since the rate of fibrosis progression increases as a function of baseline fibrosis stage [20], the above findings [49] imply that most individuals with type 2 diabetes who have stage F3 (advanced) fibrosis progress to cirrhosis within a 4 year span. Coupled with the imaging-based estimates of advanced fibrosis prevalence in type 2 diabetes (Table 1), this suggests that 10–20% of all individuals with type 2 diabetes should develop cirrhosis every 4 years. The clinical impression, however, is that the incidence of cirrhosis in type 2 diabetes is much lower. This is supported by data from the National Diabetes Register in Sweden covering almost half a million people with type 2 diabetes (90% of all individuals with type 2 diabetes in Sweden, mean age 65 years). During a follow-up period of 7.7 years, 1.3% of participants with type 2 diabetes and 0.6% of age-, sex- and county-matched controls developed severe liver disease (HR 2.28) [52]. For comparison, the risk of a cardiovascular event over 10 years in type 2 diabetes, estimated using the baseline data of these individuals, would be 10–15% in a European country with a moderate risk for CVD [53].

This prompts consideration of whether imaging biomarkers might overestimate the true prevalence of advanced fibrosis. A simple mathematical exercise shows that this is indeed a likely possibility. According to a recent meta-analysis of 1473 individuals with type 2 diabetes and MASLD, the 9.6 kPa threshold for TE, which was used most often in the studies listed in Table 1, provides a sensitivity and specificity of 77% and 71%, respectively, to identify advanced fibrosis [54]. Assuming a hypothetical advanced fibrosis prevalence of 5%, 10% or 20%, the corresponding positive predictive values (PPVs) for TE are calculated to be 12%, 23% and 40%. In other words, the false discovery rate (1−PPV) in these scenarios would be as high as 88%, 77% and 60%. This suggests that a significant proportion of individuals denoted as having advanced fibrosis based on a liver stiffness measurement (LSM) ≥9.6 kPa are likely false positives. While it is theoretically possible to back-calculate the true disease prevalence from biomarker-based estimates, variability in sensitivity and specificity across different populations (spectrum effect [55]) renders this inherently challenging. However, given that the weighted mean of prevalence estimates using the 9.6 kPa cut-off in Table 1 is 16%, the true prevalence of advanced fibrosis in type 2 diabetes may well be closer to 5%.

## How do steatosis, MASH and cirrhosis impact on mortality risk?

Among the histological features of MASLD, fibrosis consistently emerges as the best predictor of both overall and liver-related death [56]. In fact, it remained unclear for a long time whether steatosis or even MASH independently affected prognosis [51]. This uncertainty was recently addressed in the largest cohort study to date examining the impact of MASLD and its various stages on mortality risk. The study involved 10,568 participants in Sweden with biopsy-confirmed MASLD and 49,925 age-, sex- and county-matched controls, including a longer follow-up time and more deaths than all prior MASLD histology cohorts combined [57]. Over 20 years of follow-up, the presence of steatosis (HR 1.71), MASH without fibrosis (HR 2.14), non-cirrhotic fibrosis (HR 2.44) and cirrhosis (HR 3.79) significantly increased mortality risk [57]. While identical data for individuals with type 2 diabetes are unavailable, the aforementioned National Diabetes register study demonstrated a 2.29-fold higher risk of death from liver disease in diabetic individuals [52]. Additionally, there was an elevated risk of developing hepatocellular carcinoma (HCC) in both men (0.36% vs 0.10%; HR 3.67) and women (0.15% vs 0.06%; HR 2.26). These findings suggest that type 2 diabetes is associated with an approximately twofold increase in the risk of death from liver disease and an even greater relative increase in the risk of developing HCC.

## Evaluating MASLD in type 2 diabetes: what do the guidelines say?

### Evaluation of steatosis

Diagnosing MASLD necessitates clinical evidence of steatosis. The primary challenge in clinical practice, however, lies in the limited availability of methods with sufficient diagnostic accuracy. Conventional B-mode ultrasound is insensitive and detects steatosis only when it exceeds 10–20%, a level two- to four-times higher than the upper limit of normal of 5% [58]. Thus, an unremarkable hepatic ultrasound does not rule out MASLD or even advanced liver disease. More accurate modalities exist but are currently unfeasible to implement outside premier hepatology clinics due to cost and expertise requirements [58]. Risk scores incorporating clinical and laboratory data can prioritise individuals with likely steatosis but lack the precision to establish a diagnosis [58]. Consequently, MASLD mostly remains an incidental finding on abdominal imaging. Current guidelines from both the American Association for the Study of Liver Diseases (AASLD) [59] and the ADA [60] do not recommend routine screening for steatosis. A large proportion of individuals with MASLD will thus remain undiagnosed.

### Evaluation of advanced liver fibrosis

**Fig. 4:**
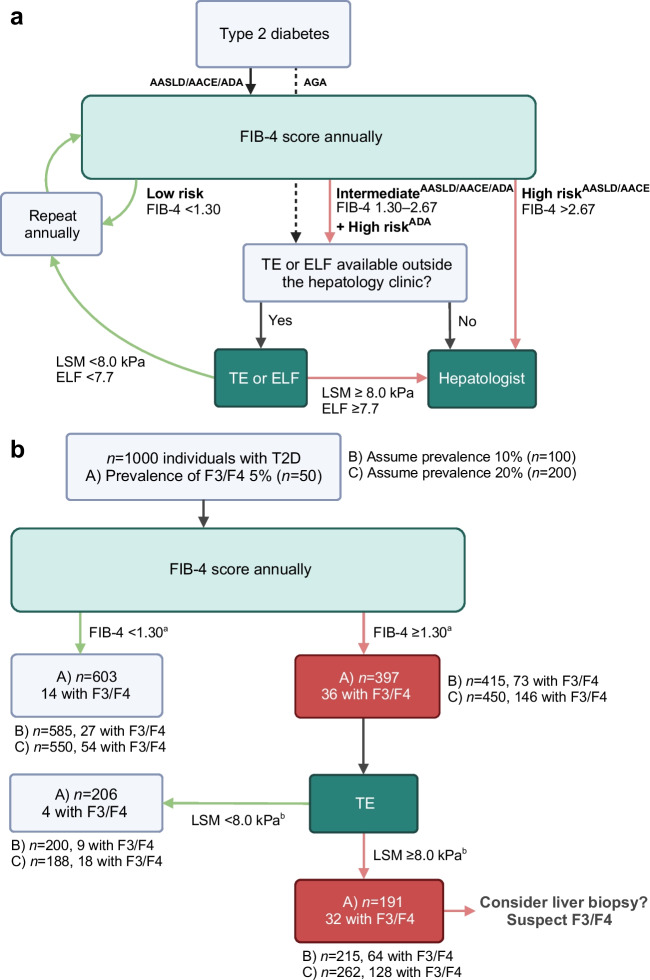
Current recommendations for screening of advanced liver fibrosis in type 2 diabetes. (**a**) Proposed algorithm to screen for advanced liver fibrosis (stage F3/F4) in type 2 diabetes. As recommended by the AASLD [59], AACE [62] and ADA [60], the FIB-4 index (a biomarker score incorporating information on age, aminotransferases and platelet count) should be calculated as the first step to rule out advanced fibrosis in type 2 diabetes. Individuals with FIB-4 <1.30 are considered low-risk and, in such cases, the FIB-4 index should be repeated annually. Individuals with FIB-4 1.30–2.67 have an intermediate risk and should undergo a secondary risk assessment using a more accurate test, which according to these guidelines is either TE (an ultrasound-based technique measuring liver stiffness based on vibrations transmitted into the liver) or the ELF (a patented blood test based on circulating extracellular matrix components). Based on the TE and/or ELF results, individuals with a high suspicion of advanced fibrosis should be referred to a hepatologist for further work-up and possibly a liver biopsy. The AASLD and AACE recommend direct referral of individuals with FIB-4 >2.67 (denoting high risk) to a hepatologist, while the ADA recommends secondary risk assessment for all individuals with FIB-4 ≥1.30. The AGA [61] recommends TE/ELF screening of all individuals with type 2 diabetes, irrespective of FIB-4 results. (**b**) Calculated patient flow when 1000 individuals with type 2 diabetes are screened according to the ADA guidelines, using the recommended cut-offs of 1.30 for FIB-4 and 8.0 kPa for TE. The boxes contain numbers of individuals based on an F3/F4 prevalence of 5% in type 2 diabetes (case A); outside the boxes are shown numbers of individuals assuming an F3/F4 prevalence of 10% (case B) or 20% (case C). ^a^The sensitivity of the 1.30 threshold for FIB-4 to diagnose F3/F4 in type 2 diabetes is 73% and specificity is 62% [54]. ^b^The sensitivity of the 8.0 kPa threshold for TE to diagnose F3/F4 in type 2 diabetes is 88% and specificity is 56% [54]. T2D, type 2 diabetes. Created with BioRender.com. This figure is available as part of a downloadable slideset

In 2023, the AASLD [59], ADA [60], and the American Gastroenterological Association (AGA) [61] published recommendations for evaluating liver disease in type 2 diabetes. A similar guideline was issued in 2022 by the American Association of Clinical Endocrinology (AACE) [62]. Given the high prevalence of MASLD in type 2 diabetes, all four societies now advocate routine screening for advanced fibrosis in this population, regardless of whether steatosis has been clinically demonstrated. This marks a departure from previous MASLD guidelines [26] and is likely influenced by recent publications suggesting a high prevalence of advanced fibrosis in type 2 diabetes (see discussion above) [59]. The recommended screening approach, summarised in Fig. 4a, involves an annual primary risk assessment using the Fibrosis-4 (FIB-4) index, followed by a secondary risk assessment using TE or the Enhanced Liver Fibrosis (ELF) test in at-risk individuals [59–62]. Notably, the AGA guidance differs significantly from those of the other societies, recommending secondary risk assessment for all individuals with type 2 diabetes irrespective of FIB-4 results [61] (Fig. 4a).

## Is universal fibrosis screening in type 2 diabetes feasible?

Concentrated efforts to identify individuals with type 2 diabetes who have clinically significant fibrosis early on are crucial. It is essential, however, to scrutinise the implications of proposed screening approaches. Fortunately for the diabetologist, the abundant literature on fibrosis biomarker performance in MASLD offers insights into the anticipated ramifications of a given screening algorithm. In individuals with type 2 diabetes and MASLD, the sensitivity and specificity of FIB-4, using the 1.30 cut-off value to rule out advanced fibrosis, is 73% and 62%, respectively [54]. The 8.0 kPa cut-off for TE has a sensitivity and specificity of 88% and 56% [54].

In Fig. 4b, we present a scenario wherein 1000 type 2 diabetic patients first undergo fibrosis screening by FIB-4 and further examination by TE in the case of FIB-4 >1.30, as suggested by the ADA [60, 62]. The figure shows examples of the expected patient flow in three cases where the prevalence of advanced fibrosis is 5%, 10% or 20% among individuals with type 2 diabetes. The low sensitivity of FIB-4 means that 27% of all patients with advanced fibrosis are missed in the initial step, having an FIB-4 value below 1.30 (Fig. 4b). While this is certainly better than flipping a coin, a more significant issue is the poor specificity, necessitating the referral of approximately 40% of all patients for TE. For a 5% prevalence of advanced fibrosis, this translates into 397 out of 1000 patients, only 9% of whom have F3/F4 (false discovery rate 91%). The use of TE decreases the number of patients requiring hepatologist referral by 206 (52%), from 397 to 191 (Fig. 4b). These patients, four of whom have advanced fibrosis, would require follow-up by annual FIB-4 unless otherwise advised. If FIB-4 remains at ≥1.30, they would likely undergo another TE examination a year later, as per guidelines. The 191 patients with LSM ≥8.0 kPa, representing 19% of the screened population, would require additional work-up by a hepatologist and possibly a liver biopsy to confirm diagnosis (Fig. 4b). Of them, however, only 32 (17%) will truly have advanced fibrosis. Thus, after screening 1000 patients with type 2 diabetes, of whom 50 have advanced fibrosis at baseline, 32 (64%) are correctly identified, 18 (36%) are missed and 159 healthy individuals (16% of the screened population and five times the number of referred cases with F3/F4) would be subjected to potentially invasive work-up at a tertiary clinic.

Considering these calculations, and the 29.7 million individuals diagnosed with type 2 diabetes in the USA alone [63], implementing the ADA algorithm [60] would mean that nearly 12 million individuals with type 2 diabetes (~40%) in the USA would require yearly TE examinations, with 5.5 million (~19%) being referred to hepatologists. As the prevalence of MASLD in type 2 diabetes is likely no higher than 60–70%, a significant proportion of those requiring hepatologist referral may not even have steatosis in the first place. These considerations imply that screening for advanced fibrosis in type 2 diabetes, as suggested by these guidelines, cannot be made with reasonable accuracy. Figure 4b also shows that this conclusion remains valid if the true prevalence of advanced fibrosis in type 2 diabetes is assumed to be 10% or even as high as 20%, which seems implausible due to reasons discussed above. Moreover, the calculations would not change appreciably if we used biomarker performance values from the largest meta-analysis of 5735 individuals with MASLD (i.e. FIB-4 [sensitivity 74%, specificity 64%] or TE [sensitivity 86%, specificity 68%] [64]), if performance values for the ELF 7.7 cut-off were used in place of TE (sensitivity 93%, specificity 34%) [65], or if individuals with FIB-4 >2.67 were referred directly to the hepatologist (as recommended by the AASLD [59] and AACE [62], data not shown).

## Does diagnosing liver fibrosis influence management of type 2 diabetes?

In the absence of specific pharmacotherapies, the pertinent question arises as to whether diagnosing advanced MASLD-fibrosis might influence type 2 diabetes management. Successful lifestyle modification is highly effective across all stages of liver disease, including compensated cirrhosis. In a paired-biopsy study involving 261 individuals with MASH (33% with type 2 diabetes), lifestyle changes resulting in ≥10% weight loss after 52 weeks resolved steatosis in 100%, MASH in 90% and regressed fibrosis in 45% of participants [66]. Similar positive outcomes were observed with bariatric surgery in a long-term follow-up study of 180 individuals with biopsy-proven MASH, wherein resolution of steatohepatitis occurred in 84% and regression of advanced fibrosis in 45% [67]. Screening for liver disease does not, therefore, change the fundamental paradigm of weight loss being the cornerstone of management in type 2 diabetes. Regardless of MASLD, most individuals with type 2 diabetes are already prime candidates for lifestyle modifications due to their high cardiovascular risk. If alerted to progressive MASLD, the diabetologist might prioritise use of glucose-lowering drugs proven effective in reversing MASH, such as glucagon-like peptide 1 (GLP-1) receptor agonists [68] and pioglitazone [69], or consider involving the bariatric surgeon. At present, the hepatologist’s primary role is to confirm diagnosis and organise surveillance and management for HCC and oesophageal varices (where appropriate). Individuals referred to academic centres may also be considered for inclusion in trials for novel pharmaceuticals. In essence, however, the most immediate effect of successful fibrosis screening is to trigger another screening programme for complications of cirrhosis. In the absence of controlled trials, whether this approach is cost-effective and translates into a survival benefit remains unclear.

## Surveying MASLD in type 2 diabetes: future directions

### A call for better biomarkers

Two major consortia, Liver Investigation: Testing Marker Utility in Steatohepatitis (LITMUS) in Europe and Non-invasive Biomarkers of Metabolic Liver Disease (NIMBLE) in the USA, will hopefully discover novel tools for evaluating MASLD. In a recent analysis of 966 LITMUS participants, only two of the 17 biomarkers tested achieved the predefined acceptable performance criterion for detecting advanced fibrosis: SomaSignal (a proteomics-based test) and ADAPT (using PRO-C3, a marker of collagen turnover) [70]. None of the biomarkers demonstrated sufficient performance in identifying MASH with clinically significant (stage ≥F2) fibrosis (‘at-risk MASH’). In a study from the NIMBLE consortium involving 1073 participants, NIS4 (a multi-marker score not included in the LITMUS study) was the only test to achieve an acceptable performance for at-risk MASH [71]. Additionally, ELF and FibroMeterVCTE (multi-marker scores) outperformed FIB-4 for all fibrosis endpoints. Both LITMUS and NIMBLE will continue gathering data in prospective cohorts, likely extending their analyses into subgroups such as individuals with type 2 diabetes. These large studies will also scrutinise the sequential use of biomarkers and explore the potential added value of genotyping individuals for common genetic risk variants of MASLD, such as *PNPLA3* I148M, as was recently suggested for individuals with type 2 diabetes and an indeterminate FIB-4 result [72]. Furthermore, additional efforts should focus on investigating ethnicity-dependent variation in biomarkers, as many tests have been extensively validated only in White populations. For example, use of different cut-offs may be appropriate for Asian individuals with MASLD [73].

### New uses for existing biomarkers

Could non-invasive tests replace the liver biopsy in MASLD risk stratification? Perhaps surprisingly, in a recent meta-analysis of 2518 individuals (46% with type 2 diabetes; 57 months of follow-up), FIB-4 and TE performed as well as histologically assessed fibrosis in predicting clinical outcomes (all-cause mortality, HCC, liver transplantation, cirrhosis complications) [74]. Accumulating evidence also shows that changes in these biomarkers over time have prognostic value. In a population-based study of 40,729 individuals, a one-unit increase in FIB-4 over a median of 2.4 years was associated with a 1.81-fold increased risk of severe liver disease during 16.2 years of follow-up [75]. In 533 individuals with advanced MASLD-fibrosis, a 20% increase in TE-LSM over a median of 3.1 years was associated with a 1.6-fold increased risk of hepatic decompensation, 1.7-fold increased risk of HCC and overall mortality, and a twofold increase in liver-related mortality, during 2.9 years of follow-up [76]. These results suggest that FIB-4 and TE not only facilitate outcome-based risk stratification in a cross-sectional manner but also have the potential to identify individuals progressing over time. Therefore, they may prove valuable for monitoring response to therapy.

### Evidence-based case-finding strategies

Rigorously conducted controlled trials are crucial to assess the feasibility, efficacy and cost-effectiveness of fibrosis case-finding strategies in MASLD. Despite extensive study on fibrosis biomarkers in various contexts, however, such data are currently lacking. Challenges in experimentation include the need for a multicentre design and a lengthy follow-up period to observe the impact on outcomes. To address these issues, the LiverScreen consortium, a European project, aims to develop an easily implementable, cost-effective and evidence-backed screening programme for liver fibrosis [77]. This initiative is designed to identify populations at risk, find the optimal tools for risk stratification, and implement a screening programme in four countries. The effectiveness of this programme will be evaluated prospectively through a randomised, controlled design against the standard of care, in 10,000 participants with a 10-year follow-up.

## Conclusions

All stages of MASLD are indisputably more common in type 2 diabetes than in non-diabetic individuals. Should all individuals with type 2 diabetes, then, be screened for advanced liver fibrosis? Based on simple biostatistical inference, the recently recommended screening pathways likely result in an unacceptably high rate of false-positive referrals to secondary tests and the hepatologist (Fig. 4b). Compounding this issue is the paucity of reliable methods by which to assess steatosis, potentially subjecting a considerable proportion of individuals with type 2 diabetes who do not have MASLD to liver biopsies—an undesirable and ethically questionable outcome. Given these facts, universal screening for liver fibrosis in type 2 diabetes should be critically re-evaluated.

We are faced with a dilemma: there is a medical need to identify individuals with type 2 diabetes and significant liver disease before hepatic decompensation occurs, but existing tools lack the specificity required. Several consortia will hopefully bridge evidence gaps and develop better methods for case-finding. In the interim, alternative approaches to fibrosis risk assessment in type 2 diabetes management could be explored, particularly for individuals at ‘intermediate risk’ (see Textbox 1: Addressing fibrosis risk in type 2 diabetes: alternative strategies). At the primary care level, biomarkers may be used more efficiently as tools for targeted implementation of weight-loss therapies and, potentially, for monitoring treatment response.

Advanced MASLD is indeed a disease where those affected likely benefit from several specialties actively working with each other. This implies that the ultimate referral channel is not only the hepatologist but also includes the diabetologist, nutritionist, bariatric surgeon and other providers with expertise in intensive lifestyle management. While many unanswered questions remain as to how patients should be best identified and managed (see Textbox 2: Unanswered questions and research needs), embracing strategies that are evidence-based and that prevent overburdening the healthcare system could garner wider acceptance among both healthcare providers and patients.

### Supplementary Information

Below is the link to the electronic supplementary material.

Supplementary file1 (PPTX 845 KB)

## Abbreviations

AACE: American Association of Clinical Endocrinology
AASLD: American Association for the Study of Liver Diseases
AGA: American Gastroenterological Association
ELF: Enhanced Liver Fibrosis
FIB-4: Fibrosis-4
GLP: Glucagon-like peptide
GPAM: Glycerol-3-phosphate acyltransferase
HCC: Hepatocellular carcinoma
^1^H-MRS: Proton magnetic resonance spectroscopy
LITMUS: Liver Investigation: Testing Marker Utility in Steatohepatitis
LSM: Liver stiffness measurement
MASH: Metabolic dysfunction-associated steatohepatitis
MASLD: Metabolic dysfunction-associated steatotic liver disease
MRE: Magnetic resonance elastography
NAFLD: Non-alcoholic fatty liver disease
NASH: Non-alcoholic steatohepatitis
NIMBLE: Non-invasive Biomarkers of Metabolic Liver Disease
PDFF: Proton density fat fraction
PPV: Positive predictive value
TE: Transient elastography
