# Supplementary material for: Surveillance of the liver in type 2 diabetes: important but unfeasible?
Source: Diabetologia. 2024 Feb 9;67(6):961–73. doi: 10.1007/s00125-024-06087-7 (PMC11058902; doi:10.1007/s00125-024-06087-7)
Supplement: Supplementary file 1 — Supplementary file1 (PPTX 845 KB) [file 125_2024_6087_MOESM1_ESM.pptx]

## Slide 1
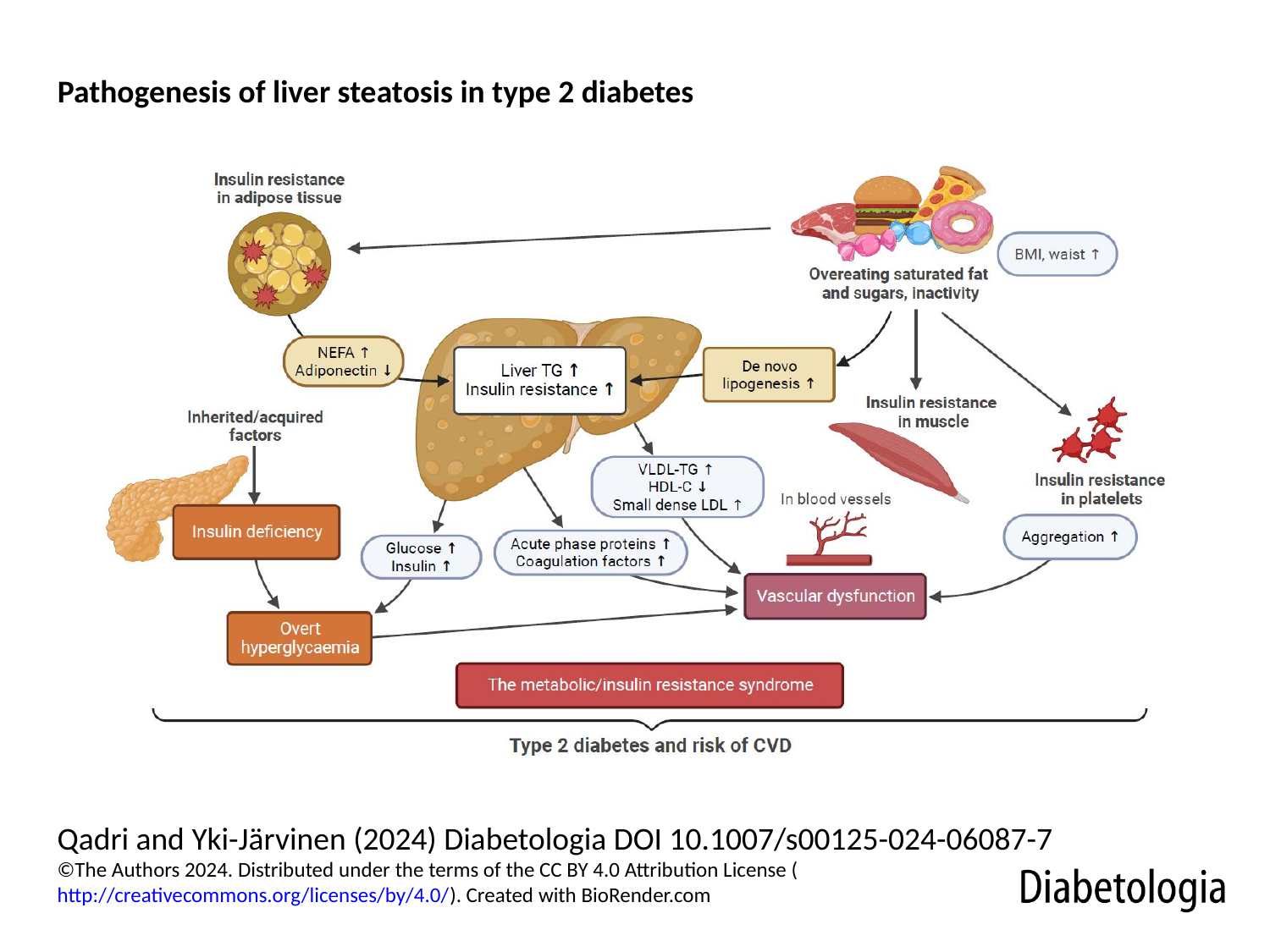

Pathogenesis of liver steatosis in type 2 diabetes
Qadri and Yki-Järvinen (2024) Diabetologia DOI 10.1007/s00125-024-06087-7
©The Authors 2024. Distributed under the terms of the CC BY 4.0 Attribution License (http://creativecommons.org/licenses/by/4.0/). Created with BioRender.com

## Slide 2
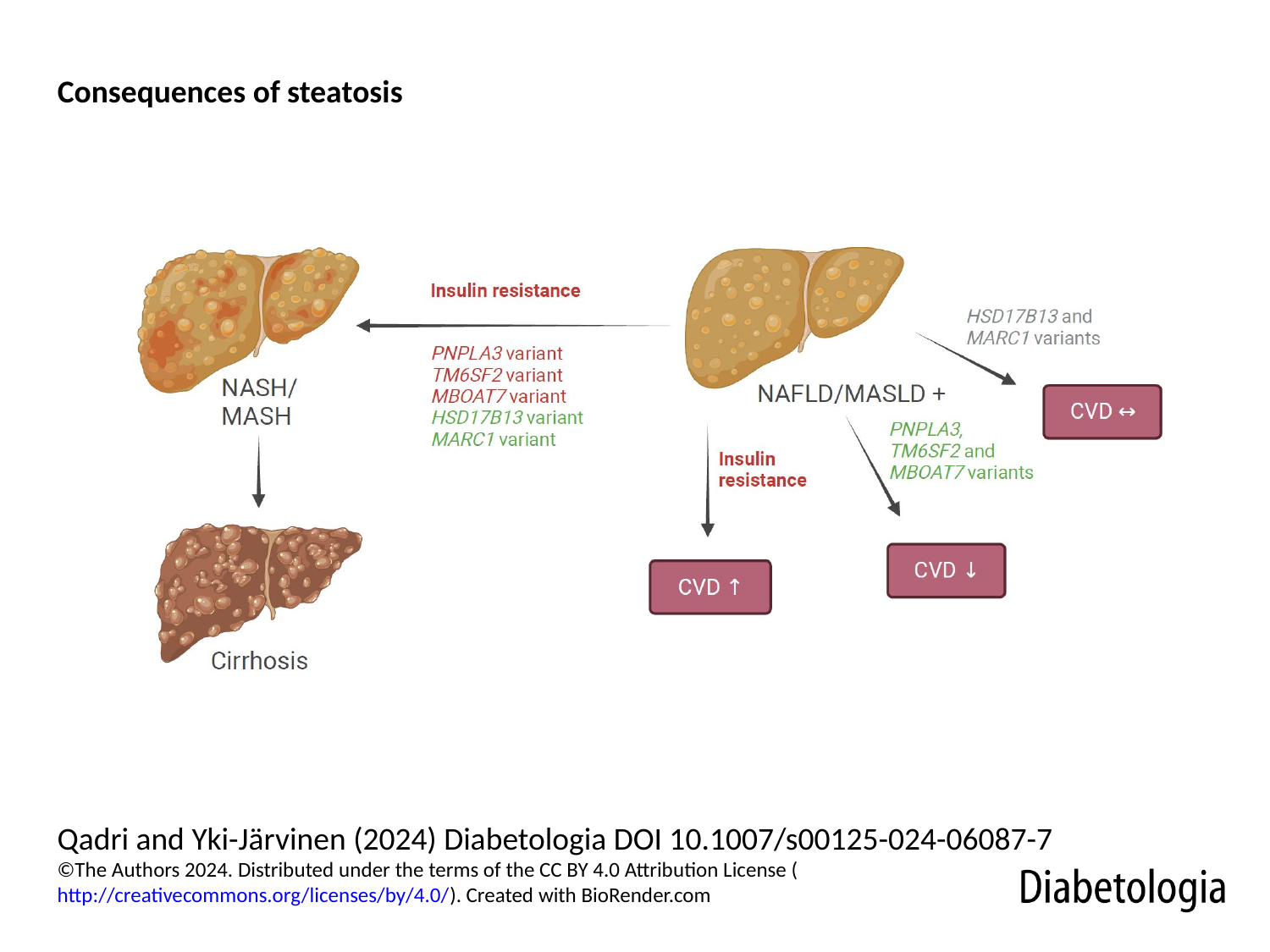

Consequences of steatosis
Qadri and Yki-Järvinen (2024) Diabetologia DOI 10.1007/s00125-024-06087-7
©The Authors 2024. Distributed under the terms of the CC BY 4.0 Attribution License (http://creativecommons.org/licenses/by/4.0/). Created with BioRender.com

## Slide 3
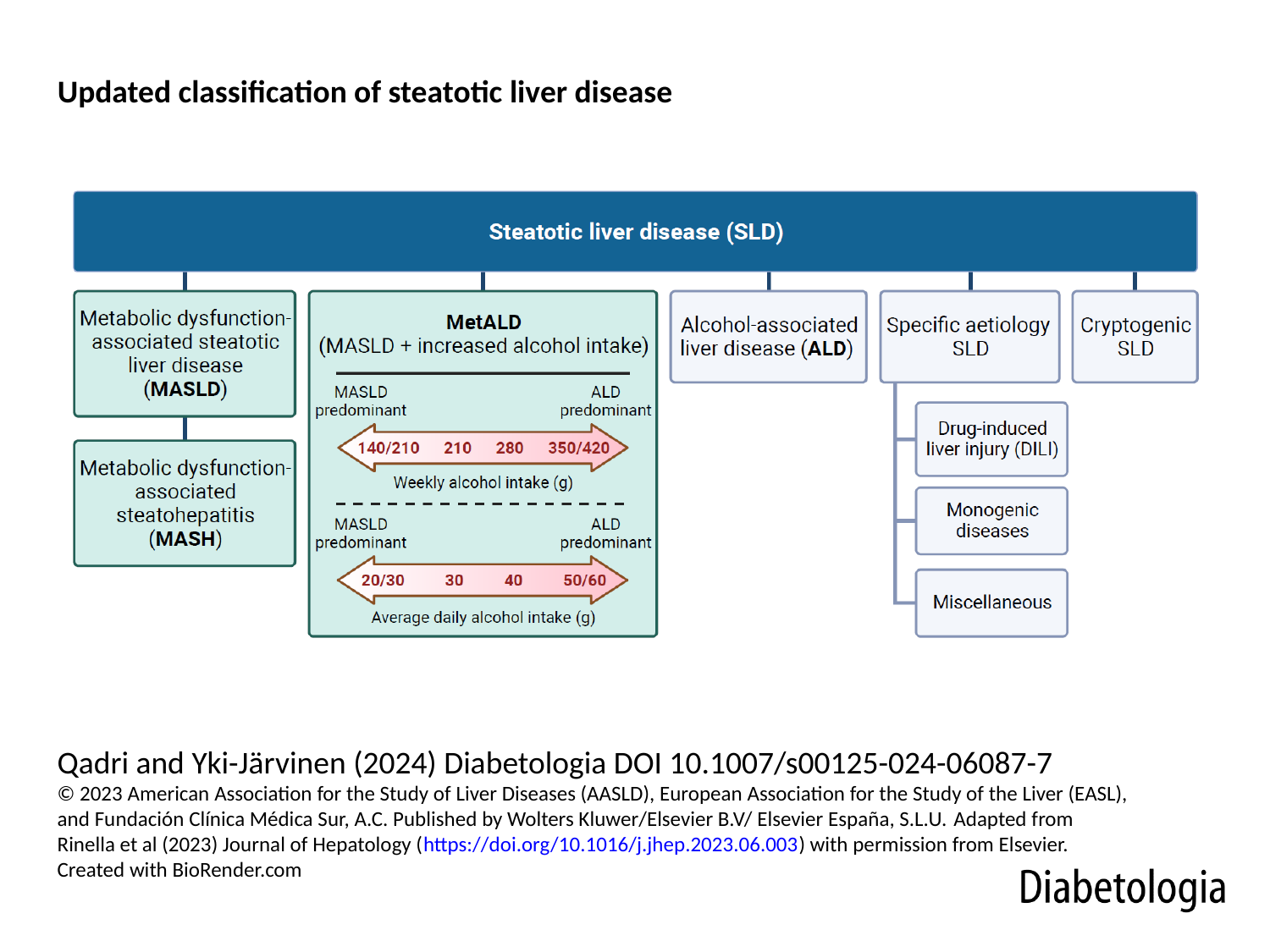

Updated classification of steatotic liver disease
Qadri and Yki-Järvinen (2024) Diabetologia DOI 10.1007/s00125-024-06087-7
© 2023 American Association for the Study of Liver Diseases (AASLD), European Association for the Study of the Liver (EASL), and Fundación Clínica Médica Sur, A.C. Published by Wolters Kluwer/Elsevier B.V/ Elsevier España, S.L.U. Adapted from Rinella et al (2023) Journal of Hepatology (https://doi.org/10.1016/j.jhep.2023.06.003) with permission from Elsevier. Created with BioRender.com

## Slide 4
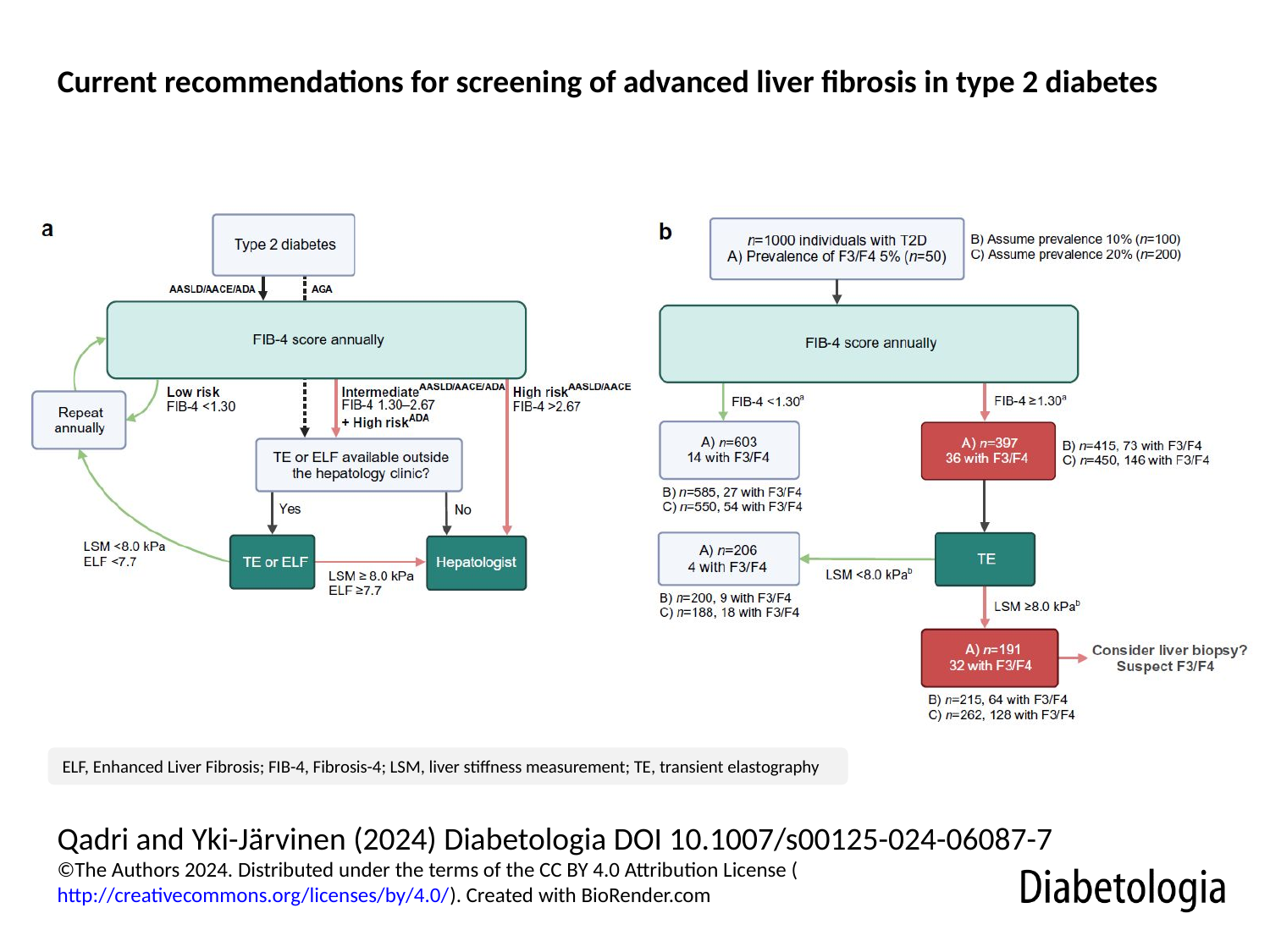

Current recommendations for screening of advanced liver fibrosis in type 2 diabetes
Qadri and Yki-Järvinen (2024) Diabetologia DOI 10.1007/s00125-024-06087-7
©The Authors 2024. Distributed under the terms of the CC BY 4.0 Attribution License (http://creativecommons.org/licenses/by/4.0/). Created with BioRender.com
ELF, Enhanced Liver Fibrosis; FIB-4, Fibrosis-4; LSM, liver stiffness measurement; TE, transient elastography
